# Supplementary material for: AffyMAPSDetector: a software tool to characterize Affymetrix GeneChip™ expression arrays with respect to SNPs
Source: BMC Bioinformatics. 2007 Jul 30;8:276. doi: 10.1186/1471-2105-8-276 (PMC1959249; doi:10.1186/1471-2105-8-276)
Supplement: Additional file 13 — AffyMAPSDetector v1 source code. AffyMAPSDetector source code. [file 1471-2105-8-276-S13.zip › AffyMAPSDetector_v1_src/doc/jwf/class-use/Wizard.html]

Uses of Class jwf.Wizard


|  |  |  |  |
| --- | --- | --- | --- |
| **Overview** \| **Package** \| **Class** \| **Use** \| **Tree** \| **Deprecated** \| **Index** \| **Help** | | |  |
| PREV   NEXT | **FRAMES**    **NO FRAMES**     **All Classes** |  |  |


---


## **Uses of Class jwf.Wizard**

|  |  |
| --- | --- |
| Packages that use Wizard | |
| **affychip.snpdetector.ui** |  |
| **affychip.snpdetector.util** |  |
| **jwf** | This package contains classes that can be used to create and run a Wizard. |

Uses of Wizard in affychip.snpdetector.ui

|  |  |
| --- | --- |
| Methods in affychip.snpdetector.ui with parameters of type Wizard | |
| `void` | `FileLoaderWizardDialog.wizardCancelled(Wizard wizard)` |
| `void` | `FileLoaderWizardDialog.wizardFinished(Wizard wizard)` |
| `void` | `FileLoaderWizardDialog.wizardPanelChanged(Wizard wizard)`             Called when a new panel has been displayed in the wizard. |

Uses of Wizard in affychip.snpdetector.util

|  |  |
| --- | --- |
| Methods in affychip.snpdetector.util with parameters of type Wizard | |
| `void` | `GeneAnnotLoaderWizard.wizardFinished(Wizard wizard)` |
| `void` | `GeneAnnotLoaderWizard.wizardCancelled(Wizard wizard)` |
| `void` | `GeneAnnotLoaderWizard.wizardPanelChanged(Wizard wizard)` |

Uses of Wizard in jwf

|  |  |
| --- | --- |
| Methods in jwf with parameters of type Wizard | |
| `void` | `WizardListener.wizardFinished(Wizard wizard)`             Called when the wizard finishes. |
| `void` | `WizardListener.wizardCancelled(Wizard wizard)`             Called when the wizard is cancelled. |
| `void` | `WizardListener.wizardPanelChanged(Wizard wizard)`             Called when a new panel has been displayed in the wizard. |
| `void` | `WizardAdapter.wizardCancelled(Wizard wizard)`             Called when the wizard is cancelled. |
| `void` | `WizardAdapter.wizardFinished(Wizard wizard)`             Called when the wizard finishes. |
| `void` | `WizardAdapter.wizardPanelChanged(Wizard wizard)`             Called when a new panel has been displayed in the wizard. |

---


|  |  |  |  |
| --- | --- | --- | --- |
| **Overview** \| **Package** \| **Class** \| **Use** \| **Tree** \| **Deprecated** \| **Index** \| **Help** | | |  |
| PREV   NEXT | **FRAMES**    **NO FRAMES**     **All Classes** |  |  |


---
